# Landscape Genomics of a Widely Distributed Snake, *Dolichophis caspius* (Gmelin, 1789) across Eastern Europe and Western Asia

**DOI:** 10.3390/genes11101218

**Published:** 2020-10-17

**Authors:** Sarita Mahtani-Williams, William Fulton, Amelie Desvars-Larrive, Sara Lado, Jean Pierre Elbers, Bálint Halpern, Dávid Herczeg, Gergely Babocsay, Boris Lauš, Zoltán Tamás Nagy, Daniel Jablonski, Oleg Kukushkin, Pablo Orozco-terWengel, Judit Vörös, Pamela Anna Burger

**Affiliations:** 1 Research Institute of Wildlife Ecology, Vetmeduni Vienna, Savoyenstrasse 1, A-1160 Vienna, Austria; saritamw@gmail.com (S.M.-W.); thomas@fultondesigns.co.uk (W.F.); Amelie.Desvars@vetmeduni.ac.at (A.D.-L.); sararibeiro.lado@gmail.com (S.L.); jean.elbers@gmail.com (J.P.E.); 2 Cardiff School of Biosciences, Cardiff University, The Sir Martin Evans Building, Museum Ave, Cardiff CF103AX, UK; orozco-terwengelpa@cardiff.ac.uk; 3 Fundación Charles Darwin, Avenida Charles Darwin s/n, Casilla 200144, Puerto Ayora EC-200350, Ecuador; 4 Institute of Food Safety, Food Technology and Veterinary Public Health, Vetmeduni Vienna, Veterinaerplatz 1, A-1210 Vienna, Austria; 5 Complexity Science Hub Vienna, Josefstädter Straße 39, A-1080 Vienna, Austria; 6 MME Birdlife Hungary, Költő utca 21., H-1121 Budapest, Hungary; balint.halpern@gmail.com (B.H.); gergely.babocsay@gmail.com (G.B.); 7 Lendület Evolutionary Ecology Research Group, Centre for Agricultural Research, Plant Protection Institute, Herman Ottó út 15., H-1022 Budapest, Hungary; herczegdavid88@gmail.com; 8 Mátra Museum of the Hungarian Natural History Museum, Kossuth Lajos utca 40., H-3200 Gyöngyös, Hungary; 9 Association HYLA, Lipocac I., No. 7, C-10000 Zagreb, Croatia; boris.laus.pmf@gmail.com; 10 Independent Researcher, Hielscherstraße 25, D-13158 Berlin, Germany; lustimaci@yahoo.com; 11 Department of Zoology, Comenius University in Bratislava, Ilkovičova 6, Mlynská Dolina, S-84215 Bratislava, Slovakia; daniel.jablonski@balcanica.cz; 12 Department of Biodiversity Studies and Ecological Monitoring, T. I. Vyazemsky Karadag Scientific Station–Nature Reserve–Branch of Institute of Biology of the Southern Seas of the Russian Academy of Sciences, Nauki Street 24, R-298188 Theodosia, Crimea; vipera_kuk@ukr.net; 13 Department of Herpetology, Zoological Institute of the Russian Academy of Sciences, Universitetskaya Embankment 1, R-199034 Saint Petersburg, Russia; 14 Department of Zoology, Hungarian Natural History Museum, Baross u. 13., H-1088 Budapest, Hungary; 15 Molecular Taxonomy Laboratory, Hungarian Natural History Museum, Ludovika tér 2-6., H-1083 Budapest, Hungary

**Keywords:** adaptive evolution, Caspian whipsnake, ddRAD, environmental correlates, genetic diversity

## Abstract

Across the distribution of the Caspian whipsnake (*Dolichophis caspius*), populations have become increasingly disconnected due to habitat alteration. To understand population dynamics and this widespread but locally endangered snake’s adaptive potential, we investigated population structure, admixture, and effective migration patterns. We took a landscape-genomic approach to identify selected genotypes associated with environmental variables relevant to *D. caspius*. With double-digest restriction-site associated DNA (ddRAD) sequencing of 53 samples resulting in 17,518 single nucleotide polymorphisms (SNPs), we identified 8 clusters within *D. caspius* reflecting complex evolutionary patterns of the species. Estimated Effective Migration Surfaces (EEMS) revealed higher-than-average gene flow in most of the Balkan Peninsula and lower-than-average gene flow along the middle section of the Danube River. Landscape genomic analysis identified 751 selected genotypes correlated with 7 climatic variables. Isothermality correlated with the highest number of selected genotypes (478) located in 41 genes, followed by annual range (127) and annual mean temperature (87). We conclude that environmental variables, especially the day-to-night temperature oscillation in comparison to the summer-to-winter oscillation, may have an important role in the distribution and adaptation of *D. caspius*.

## 1. Introduction

Adaptation to a changing environment is a growing challenge that populations face in the era of “Anthropocene”, marked by significant human impact on the Earth’s geology and ecosystems, including human-mediated climate change [1]. Populations capable of rapid adaptation to novel selection pressures are more likely to survive. Rapid evolution is fuelled by standing genetic variation, rather than the emergence of de novo mutations [2,3]. Therefore, maintaining standing genetic variation is crucial for populations to adapt in changing environments. To predict the ability of populations to respond to environmental change, we need to look for the genetic basis of adaptation and understand the mechanisms of adaptive responses to specific environmental and/or anthropogenic challenges [2,4,5]. Understanding such microevolutionary processes requires genome-wide data, adequate geographical sampling, and a landscape genomic approach [6,7,8].

The Caspian whipsnake, *Dolichophis caspius* (Gmelin, 1789), is a large-bodied colubrid species with a distribution covering large parts of eastern Europe including the Balkans, Turkey, and regions around the Caspian and the Black Sea [9,10,11,12]. The species reaches the north-western edge of its distribution range in Hungary, where it occurs in isolated populations located mainly along the Danube River and southern Hungary [11]. *Dolichophis caspius* also inhabits the Croatian islands, Olib and Lastovo [13], where they are suspected of having been introduced by humans. The Caspian whipsnake inhabits dry steppe and Mediterranean habitats, including loess grasslands and scrubby rocky outcrops [14,15,16,17,18]. It is a thermophile and strictly diurnal snake [14]. According to its present patchy distribution in Hungary [15,19], its dispersal seems to be hindered either by heavily forested areas or extensively cultivated croplands. Our knowledge is scarce on how the current and future change in climate affects this species in its current habitats and its future range shift.

In the northern parts of its distribution range, the Caspian whipsnake is under strict protection in Croatia [13], Hungary [20], Ukraine [21], and Romania [16]. Its conservation status is of least concern, according to the International Union for Conservation of Nature (IUCN) European Red List of Reptiles [22].

While evolutionary forces such as gene flow [23], founder effects [24], genetic drift [25], and natural selection [26] have been frequently studied in snakes, locus-specific effects associated with genes under selection and landscape genomics have not yet been investigated in *D. caspius*. Genetic studies of *D. caspius* have been scarce, and the existing morphological studies focused either on separating *D. caspius* from the *jugularis* species complex [27] or were geographically too restricted to allow evaluation of within species geographical variation across the entire distribution range [28,29]. The only spatially large-scale study focused on DNA sequences of the mitochondrial cytochrome *b* gene and was a phylogeographic analysis of samples from South-East Europe (Balkan Peninsula, Hungary, and Romania) and Frontier Asia [11]. Results revealed two haplotype groups, dividing the populations into an Anatolian eastern and a western European lineage differentiated in the Pleistocene, corresponding with the Eurasian and Anatolian tectonic plates currently separated by the Aegean Sea and the Bosporus Strait.

Genetic variation is essential for a population to respond to changing environments (e.g., urbanization or climate change) and to emerging infectious diseases (e.g., snake fungal disease [30]) effectively. Investigating genetic variability and population connectivity to identify genetically distinct and isolated populations and to develop species management and conservation programs is, therefore, important to reduce extinction risks [31,32]. The overall goal of our study was to understand the population dynamics and potential for environmental adaptation in a widely distributed Old World snake, *D. caspius*, as a representative organism for reptiles across eastern Europe and western Asia. To investigate local adaptation, we investigated *D. caspius* (i) population structure, (ii) genetic diversity, and (iii) adaptive evolution to local habitat types throughout its distribution, applying genome-wide double-digest restriction-site associated DNA sequencing (ddRAD-seq) data and a landscape-genomic approach.

## 2. Materials and Methods

### 2.1. Samples and DNA Extraction

**Figure 1:**
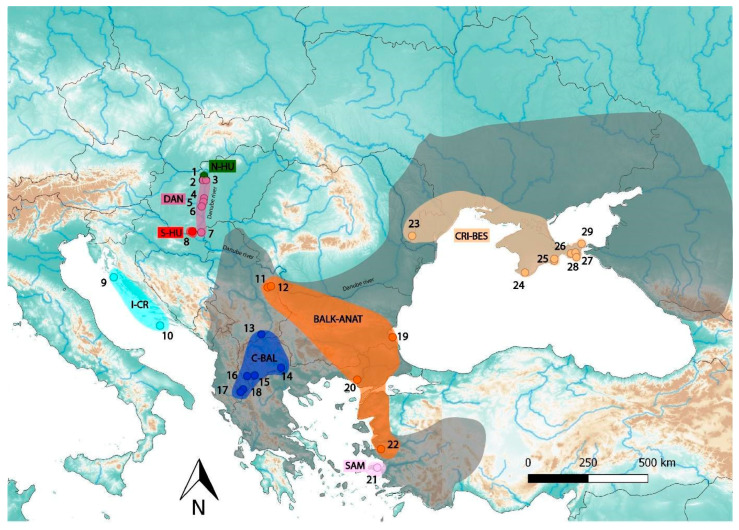
Map of the sampling locations. *D. caspius* populations are labeled corresponding to Table 1. Different colors represent eight genetic populations identified by phylogenetic and Principal Component Analysis. The dark shaded area shows the approximate distribution of *D. caspius* following Sillero et al. [33], slightly modified with the authors’ personal observations.

Extracted from carcass tissue, shed skin and blood, and buccal swabs originating from 8 countries, 124 DNA samples were collected for this study (Figure 1 and Table 1) and deposited at the Collection of Genetic Resources of the Hungarian Natural History Museum in Budapest. Spatial data (longitude and latitude in decimal degrees) were recorded using a GPS device for each sampled individual. We extracted DNA from buccal swabs using the blackPREP Swab DNA Kit according to kit protocols (Analytik Jena AG, Jena, Germany). For shed skin and carcass (liver and muscle) samples, the DNEasy Blood and Tissue Kit (Qiagen, Hilden, Germany) was applied following manufacturer’s instructions but adding 30 μL of Proteinase K (20 mg/μL) to increase lysis efficacy. Prior to the extraction, skin samples were soaked in water for 24 h at room temperature and manually sliced into fragments of ~5 mm to facilitate enzymatic lysis. Samples’ DNA quality (measured by absorbance) was determined using a NanoDrop 2000 spectrophotometer (Thermo Scientific, Waltham, MA, USA). The 96 best extractions were selected based on a concentration threshold of 10 ng/μL and 260/280 ratio values > 1.85 and used for ddRAD sequencing.

### 2.2. Double-Digest Restriction-Site Associated DNA (ddRAD) Sequencing and SNP Filtering

DNA concentrations were normalized prior to the library preparation to ensure homogeneous sequencing results followed by double restriction enzyme digestion with *SphI* and *HindIII*. The ddRAD library preparation following Peterson et al. [34] and sequencing was performed at the Institute of Applied Genomics (IGA Technology Services, Udine, Italy) on an Illumina HiSeq2500 with 125 bp paired-end reads across multiple lanes and multiplexed with 80 bp sequencing adaptors. Forward and reverse reads were concatenated, demultiplexed to separate each individual by barcodes, filtered to remove low-quality reads and sequencing adapters, and trimmed to 110 bp with the process_radtags tool from Stacks v. 1.35 [35] using default parameters. For quality trimming, process_radtags applied a sliding window analysis using the 15% of read length from 5′ to 3′ of each read. If the average quality of a window dropped below a phred score of 10, the read was discarded. SNPs were called from the resulting reads using the ustacks-cstacks-sstacks-rxstacks-populations pipeline within Stacks v. 1.45 and keeping only one random SNP per RAD locus, which resulted in 24,497 SNPs. Sequence coverage depth of SNPs for each individual was calculated using VCFtools 0.1.16 (see https://doi.org/10.5061/dryad.mgqnk98xmfor description of the Stacks pipeline).

The functions geno and mind within Plink 1.07 [36] were used to remove SNPs with missing call rates exceeding 25% and to exclude individuals with over 25% missing genotypes (*n* = 19) from the dataset. SNPs were then filtered for 1% minor allele frequency (maf). Duplicated samples and highly-related individuals (parent-offspring or siblings) were identified by calculating pairwise identity-by-state (IBS) values using the distance matrix function within Plink. From the resulting matrix, pairs of individuals with an allele identity (IBS) higher than 0.95 (i.e., a coefficient of inbreeding (COI) of 5%, interpreted as a pairwise match [36]) were reduced to 1 sample per pair to avoid biasing allele-frequency estimations. The removed samples included a total of 11 samples from Olib and Lastovo islands, Croatia (IBS > 0.98); 1 from Paks, central Hungary (IBS > 0.97); 2 from Pesthidegkút, northern Hungary (IBS > 0.95); and 9 from Villány Hills, southern Hungary (IBS > 0.95). As the samples in Hungary were collected in the course of regular monitoring in the frame of the *D. caspius* species conservation program, the high number of samples with IBS > 0.95 likely reflect the recapture of snakes or re-sampling of shed skins. In the Croatian islands, the snakes were surveyed annually, individually recognizing each specimen, thus re-sampling or recapture can be excluded. Despite the removal of samples, the remaining dataset included individuals from each sampled locality (Table 1). The final dataset for downstream analysis consisted of 53 individuals and 17,518 SNPs with a 43-fold average coverage depth over all individuals (ranging from 8- to 109-fold; Table S1).

### 2.3. Population Summary Statistics, Structure and Differentiation

The package pegas [37] within RStudio [38] was used to calculate overall expected (*H*_E_) and observed heterozygosity (*H*_O_), which were compared using a parametric Welch’s t-test implemented in R statistical environment v.3.5.1. [39] using the *t*-test function. We explored the initial population structure with a Principal Component Analysis (PCA) implemented in Adegenet v.2.1.0 [40]. Phylogenetic relationships between individuals were visualized in a NeighbourNet network implemented in Splitstree v.4.10 [41] based on a pairwise genetic distance matrix calculated with Plink v.1.07. To further identify the number of ancestral populations (K) and potential admixture in *D. caspius* populations—Admixture v.1.3 [42] was run, testing for values of K between 2 and 9. We adopted the 5-fold Cross Validation (CV) error method to determine the best number of clusters (K) following the developers’ recommendations. An analysis of molecular variance (AMOVA) was then carried out within Arlequin v.3.5.2. [43] to assess how genetic variation was partitioned within the hierarchical population structure (clusters) of *D. caspius*. Arlequin was also used to estimate hierarchical *F*-statistics as well as nucleotide diversity (π) within each subpopulation using 10,000 permutations to determine significance in all Arlequin based analyses. Subpopulations containing fewer than 4 individuals (i.e., I-CR, BALK-ANAT, and DAN) were excluded from the analysis.

### 2.4. Estimating Effective Migration Surfaces (EEMS)

The software EEMS (Estimated Effective Migration Surfaces) [44] was used to estimate effective migration patterns between samples for 2 different sets of individuals. For the first set, we used the total dataset of 53 samples and selected a polygon accounting for the known species distribution using a grid of 600 demes. This was done in order to obtain an approximation of the migration patterns across the species distribution range. Second, we used a smaller dataset (34 samples), including samples from Hungary, southern Serbia, North Macedonia, and southern Albania using a grid of 500 demes. This was carried out to obtain an approximation of migration patterns in central Europe, where the distribution of the species was the most scattered but the best sampled. EEMS’ Markov chain Monte Carlo (MCMC) algorithm was run for 10,000,000 steps discarding the first 50% as burn-in and saving every 49,995th step to estimate the migration parameters. Two independent runs were carried out to assess the convergence of the MCMC runs. The habitat polygon per dataset was obtained using Google Maps API v3 Tool (http://www.birdtheme.org/useful/v3tool.html), and results were plotted using the R package rEEMSplots as suggested in Petkova et al. [44].

### 2.5. Landscape Genomic Analysis

#### 2.5.1. Selection of Environmental Variables

We used current climate data (1970–2000) from the WorldClim Version 2 dataset [45]. The environmental variables encompassed average monthly mean and maximum temperature (°C), precipitation (mm), wind speed (m·s^−1^), water vapor pressure (kPa), solar radiation (kJ·m^−2^·day^−1^), and the 19 WorldClim bioclimatic variables at 30 arc-second resolutions (Table S2). The values of the WorldClim variables at the sample locations were extracted from the raster dataset (extract function in the R package raster).

#### 2.5.2. Correlation Analysis of Environmental Variables

Because multicollinearity of variables can result in erroneous modeling [46], we removed highly correlated environmental predictors. The variance inflation factor (VIF) was calculated for each environmental variable to detect the presence of collinearity [47] with a cut-off value of VIF < 5 following Stucki et al. [48]. The VIF was calculated using the R package fmsb [49]. To determine which environmental variables contributed most to the overall variation in the individuals’ distribution, we run a PCA considering the 7 selected variables and the 53 individuals using the prcomp function in R software. Data were scaled to account for differences in units among each variable.

#### 2.5.3. Samßada Analysis

Samßada (v. 0.6.0) modeled the probability of a genotype occurrence in an individual according to its habitat’s environmental composition using logistic regressions while taking population structure into account [48]. First, the null model of dimension *P*, including the population variables and genotype data, was computed for each genotype. To describe the population variables, we used the eigenvalues of the PCA’s first 3 principal components (PC1-3), explaining 41.05% of the total variation in the *D. caspius* dataset. The effect of each environmental variable was tested by adding one environmental variable at a time to the population variables (dimension *P + 1*) and assessing which of the 2 models (without or with the environmental variable) was more likely.

For each tested model, Samßada created an output file, which contained the model parameters for each genotype, including log-likelihood values. Following the Samßada user manual’s instructions, we calculated a G-score for each tested model from the log-likelihoods of dimensions *P* and *P + 1* (G = 2 × (l_P+1_ − l_P_)). Finally, *p*-values (*P*) were calculated and corrected for multiple comparisons using the Benjamini and Hochberg procedure [50]. The workflow diagram (Supplementary Figure S1) and detailed bioinformatics analyses are provided in the Supplementary Materials. The ddRAD sequence reads adjacent to the SNPs identified to be under selection were mapped with BLASTN v. 2.2.31+ [51] to the *Thamnophis sirtalis* 6.0 genome assembly (GenBank: GCF_001077635.1). Protein-coding genes containing SNPs were determined by using the NCBI Eukaryotic Genome Annotation pipeline’s annotation release 100 for the *T. sirtalis* genome, which was based on sequence homology between a multitude of taxa (https://www.ncbi.nlm.nih.gov/genome/annotation_euk/process/) and also ab initio models and thus produced in silico functional annotations also for more distantly related (non-model) organisms. Similar annotation pipelines have been applied to a wide range of genetic distances, from distant members of the same clade to individualized assemblies of the same species [52]. Described functions of protein-coding genes were inferred from GeneCards^®^ database (www.genecards.org).

## 3. Results

### 3.1. Population Structure and Genetic Differentiation among D. caspius Populations across the Entire Distribution Range

#### 3.1.1. Overall Genetic Diversity

The expected heterozygosity (*H*_E_) estimated across all 53 individuals was significantly higher than the observed heterozygosity (*H*_O_) under Hardy–Weinberg equilibrium: 0.204 (±0.177) and 0.107 (±0.106), respectively. The overall average inbreeding coefficient across all individuals was *F*_IS_ = 0.132.

#### 3.1.2. Population Structure and Admixture

**Figure 2:**
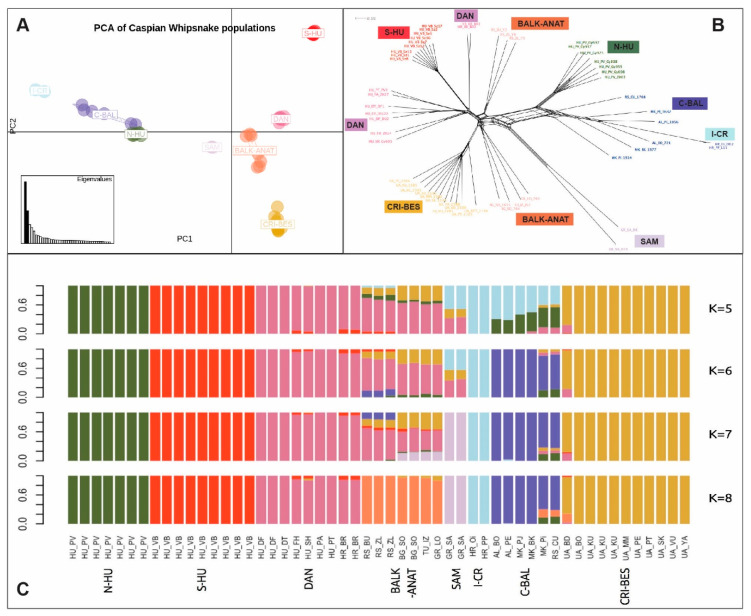
(**A**) Principal Component Analysis of 53 *D. caspius* individuals. The first and second principal components explain 22.3% and 11.9% of the genetic variation in the dataset, respectively. (**B**) Unrooted NeighbourNet network tree displaying relationships among the 53 *D. caspius* individuals based on genetic distance. (**C**) Admixture barplots for K = 5–8 in 53 individuals. Populations are labeled, corresponding to Table 1.

We visualized population structure in *D. caspius* with a PCA using pairwise genetic distances calculated on individual allele frequencies. The amount of the genetic variation represented by the first (22.3%), second (11.9%), and third (6.9%) principal components clustered the *D. caspius* in eight different groups (Figure 2A), with PC (principal component)1 separating populations from southern Hungary (S-HU), the Danube region (DAN) of Hungary and Croatia, Balkan-Anatolia, including western Turkey, Bulgaria, Greece and Serbia (BALK-ANAT), the Greek island Samos (SAM), and the Northern Black Sea region including the Crimean Peninsula and Bessarabia in Ukraine (CRI-BES) from those in northern Hungary (N-HU), Dalmatian Archipelago of Croatia (I-CR), and the central Balkans in the limits of southern Albania and Republic of North Macedonia (C-BAL). Notably, snakes from N-HU seemed to be genetically more related to the individuals from C-BAL than to their geographically closer neighbors from S-HU. We identified a similar structure in the phylogenetic NeighbourNet network, where the evolutionarily-close relationship between the I-CR, N-HU, and C-BAL population was reflected (Figure 2B). The snake individuals from SAM clustered apart from the BALK-ANAT population.

Further analyses of *D. caspius* population structure using Admixture identified the best ancestral model to be K = 5 (with the lowest Cross Validation error CV = 0.45). These results indicated that most admixture was observed in the populations from C-BAL and BALK-ANAT. C-BAL showed substantial admixture between the N-HU and I-CR populations and a limited amount from the DAN and CRI-BES populations. BALK-ANAT harbored a mixed genetic make-up sharing genetic ancestry with the DAN and CRI-BES populations and to a limited extent also with N-HU, SAM, and S-HU. The results for K = 6 or 7 were similar to those for K = 5 but with relatively small changes in the proportions of admixture. However, increasing the number of potential ancestral populations to K = 8 resulted in the same eight groups identified by the PCA analysis (Figure 1 and Figure 2C). Further higher numbers of K did not reveal finer scale structure (results not presented). Incorporating all coherent information from the analyses above, we defined eight current populations in the European and Asian *D. caspius* and used this structural grouping in subsequent analyses (Table 1).

#### 3.1.3. Population Differentiation

AMOVA of the total dataset subdivided into eight populations showed that the majority of genetic variation was detected among all individuals (50.8%), followed by variation between populations (41.5%) and then among individuals within populations (7.7%). The mean nucleotide diversity (π) within the different populations ranged from 0.067 (S-HU) to 0.168 (BALK-ANAT) (Table S3. Population inbreeding coefficients (*F*_IS_) were significantly different than zero only in the DAN population (*F*_IS_ = 0.44; *p* < 0.001) and in the C-BAL population (*F*_IS_ = 0.121; *p* < 0.04), albeit the latter was small (Table S3. Pairwise *F*_ST_ values ranged between 0.132 and 0.595 (*p* < 0.05), with the highest differentiation levels observed between S-HU and N-HU population, followed by S-HU and C-BAL, while moderate pairwise genetic distances were found between the N-HU and DAN, BALK-ANAT and C-BAL populations (*F*_ST_ = 0.261–0.420). The lowest differentiation was found between BALK-ANAT and DAN or CRI-BES (Table 2).

### 3.2. Estimated Effective Migration among D. caspius Populations

**Figure 3:**
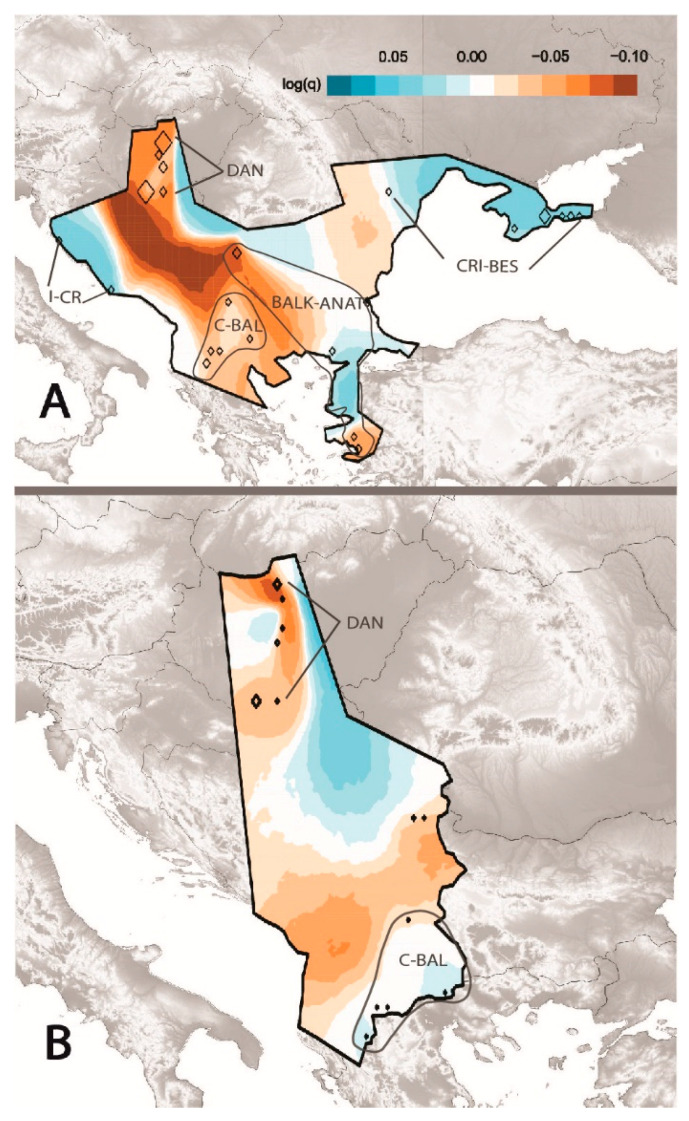
Effective migration patterns from the total dataset (**A**) and from Central Europe (**B**). Diamond-shaped symbols represent samples, and size is proportional to the number of individuals. Higher-than-average (blue) and lower-than-average (brown) effective migration rates between sampling locations are shown.

Looking at the complete dataset, we observed no large migration corridor across the distribution area (Figure 3A). However, we observed small patches of possible migration corridors with effective migration rates higher than the overall mean. Higher-than-average effective migration rates were observed within CRI-BES, and between the I-CR. On the other hand, we observed a migration barrier that coincided with the Dinaric Alps. Lower-than-average migration was observed along the Danube (DAN), within the C-BAL clade, and between C-BAL and BALK-ANAT clades. When restricting the analysis to comprise only of the samples from the Carpathian Basin and from the Northern and Central Balkans, the results suggested similar patterns (Figure 3B). However, lower-than-average migration became more pronounced along the DAN region, and higher-than-average to average estimated migration surfaces appeared within the C-BAL group.

### 3.3. Relationship between Genotypes and Environmental Variables

We used a landscape-genomic approach implemented in Samßada to investigate potential correlation between genotype and environmental data across the distribution range of *D. caspius* in eastern Europe and western Asia. From a set of 24 environmental variables (WorldClim), we selected the least correlated ones (*n* = 7; VIF < 5; Table S2) for our landscape genomics model: Average wind speed in April (wind04), average annual mean temperature (bio01), average isothermality (bio03), average temperature annual range (bio07), average mean temperature of the wettest quarter (bio08), average annual precipitation (bio12), and average precipitation of the driest quarter (bio17) [53] (Table S4). We visualized the environmental data structure with a PCA biplot using the seven explanatory environmental variables calculated for the 53 individuals (Figure S2). The first three components of the PCA explained 82.3% of the variance and a good clustering of the individuals was obtained according to the population of origin. PC1 explained 34.8% of the variance and was mostly correlated with bio03 (Isothermality) (−0.78) and bio12 (annual precipitation) (−0.70). PC2 explained 28.2% of the variance and was mostly correlated with wind04 (wind in April) (−0.72), bio01 (annual mean temperature) (−0.70) and bio17 (precipitation of driest quarter) (0.64), and PC3 was mostly correlated with bio17 (0.62). The variables bio03 and bio12 on one side and bio07 and bio17 on the other side were positively correlated, whereas bio07/bio17 and bio01 loaded strongly in opposite directions. Individuals from C-BAL showed high values for bio03 and bio12, whereas individuals from DAN and N-HU were separated from the other populations by bio08 (mean temperature of wettest quarter). Individuals from CR-BES were mostly separated by wind04, while those from BALK-ANAT and I-CR were mostly characterized by bio01.

We aimed to detect the correlation between genotypes and environmental variables whilst taking population structure into account by including population differentiation as a covariate in the analysis. This should enable us to identify signatures of selection that appear on top of the observed population structure, which was represented by the first three PCs explaining 41.05% of the variation in *D. caspius*. A total of 751 genotypes were significantly (*p* < 0.01) associated with one of the seven environmental variables, and of these 65 were located within coding regions predicted in *T. sirtalis* (Figure S2). Average isothermality correlated to the highest number of genotypes and genes (478|41, respectively), followed by the average temperature annual range (127|13), the average annual mean temperature (87|5), average wind speed in April (29|4), and the precipitation of the driest quarter (22|1). The environmental variables showing the least number of genotypic associations were the mean temperature of the wettest quarter (6|1) and the annual precipitation (2|0). For those environmental variables with the highest number of associated genes, we screened their described functions, e.g., genes significantly associated with isothermality were allocated to functions like neuronal development and differentiation, signal transduction (ACVR1B, ADGPL1, DOCK7, NFASC), as well as encoding proteins of calcium channel subunits (CACN11, CACNA1S, RYR2, TCF7L2) and of the RET signaling pathway (CNKR1). One gene (PAM) encoding a multifunctional protein, which catalyzes the conversion of neuroendocrine peptides, was associated with two environmental variables: Isothermality and average temperature annual range. The complete list of genes associated with environmental variables in different *D. caspius* individuals is given in Figure S2.

## 4. Discussion

Rapid climate change and habitat fragmentation can have negative impacts on populations and be responsible for significant biodiversity loss, making them important concerns in the field of conservation biology [54,55]. In certain regions of the Central-Eastern European range, *D. caspius* populations have increasingly become disconnected due to habitat degradation (intensive agriculture in the originally steppe areas) and alterations. To understand the population dynamics and adaptation potential of this widespread but locally endangered snake, we investigated population structure, admixture, and effective migration patterns across its distribution range. Moreover, we also implemented a landscape-genomic approach to identify genotypes associated with environmental variables across the species distribution range.

### 4.1. Structured Population and Effective Migration Rates in D. caspius across Two Continents

Genetic structuring of reptiles in Europe follows a well-known North-South pattern, with the highest genetic differentiation observed in southern Europe and the Mediterranean peninsulas, while the lowest genetic differentiation is observed in northern Europe [56]. The ‘southern richness’ is explained by the location of former refugia during several glacial cycles, and the lower levels of genetic diversity in the northern range can be explained by the rapid postglacial range expansion and loss of diversity on the way [57].

Our analyses identified eight distinct genetic clusters across the entire distribution of *D. caspius* (Figure 1 and Figure 2). Four of them contained specimens from the Balkans and Aegean coast of Asia Minor Peninsula (C-BAL, BALK-ANAT, SAM and I-CR), one contained specimens from the Northern Black Sea region (CRI-BES), and three clusters included individuals from the Carpathian Basin (DAN, N-HU, and S-HU). While the high genetic differentiation among *D. caspius* populations in the southern regions was expected considering the putative glacial refugia located in the southern peninsulas for nearly the whole European fauna [58,59], the significant structuring at the north-western limit of the distribution in the Carpathian Basin was striking. The differentiation between DAN, S-HU, and N-HU clades could be traced back to different origins. During the last 200 years, the once continuous dolomite and loess walls along the Danube River suffered extreme habitat fragmentation induced mostly by urban sprawl [19,60]. This fragmentation is likely to have caused the isolation of *D. caspius* into small patchy populations and could explain the highest level of inbreeding observed in the DAN clade (*F*_IS_ = 0.446; Table S3), and the comparatively higher inbreeding coefficient than in the rest of the populations (*F*_IS_ < 0.121; Table S3).

Surprisingly, the northernmost population, N-HU was more closely related to individuals from North Macedonia (C-BAL) and showed genetic separation from the spatially closest Hungarian populations (i.e., DAN and S-HU; Figure 2 and Table 2). This raises the possibility of a human translocation, most likely from the Balkan Peninsula. Alternatively, it could be a remnant population from a previous colonization event. In contrast, the S-HU group shows the smallest genetic distance with the spatially nearest DAN group. This is concordant with the findings of Nagy et al. [11], who suggested that the Villány Hills might have been an ideal area for the survival of *D. caspius* during the last glaciation’s moderately cold periods and further supporting the microrefugia hypothesis of European reptiles [56,61]. The human introduction might explain the genetic origin of I-CR group, which seemingly has diverged from the C-BAL group in the last few hundred years, as suggested by the few differences observed between the two samples from distant geographical locations (>500 km apart) analyzed here (Figure 2). Furthermore, the first sightings of *D. caspius* in that region were first documented in 1902 [62]. However, it is possible that these populations arrived at the Croatian islands much earlier but eluded detection due to their small population size.

The Estimated Effective Migration Surfaces confirmed the observed pattern of the clustering analyses (Figure 3). Higher migration rates in the northern Black Sea region (CRI-BES) suggested a larger and more continuous population. Despite the possible migration corridor along the Danube River, lower migration in the DAN group pointed out the discontinuity and isolation of populations. Lower-than-average migration within the C-BAL group and between C-BAL and BALK-ANAT suggest geographic barriers of gene flow, such as the Dinaric Alps separating the continental Balkan Peninsula from the Adriatic Sea.

Snake species with similar geographic distribution show only a partially concordant pattern. The eastern genetic clade of the Aesculapian snake (*Zamenis longissimus*) occupied northern central Europe from a Balkan refugium only during the Holocene climatic optimum, and has low genetic variation across the whole area [63,64,65], except for a distinct clade in Greece and another one east of the Black Sea. A single genetic lineage of the smooth snake (*Coronella austriaca*) dominates central Europe; another clade occupies the Balkans, while high diversity can be found in Anatolia and Transcaucasia [66]. The common grass snake (*Natrix natrix*) shows a complex genetic pattern across Central Europe and the Balkans. One genetic lineage distributed from Scandinavia to the Balkan Peninsula survived glaciations in two distinct refugia in the southern Balkan Peninsula and in Central Europe, while the second genetic lineage colonized Central Europe recently from a structured refugia in the Balkan Peninsula [61].

### 4.2. Environmental Adaptation in D. caspius

Determining genomic diversity and adaptive selection relative to a species’ environment is important for understanding species resilience and adaptive potential [67]. With a landscape genomics approach, we sought to resolve the influence of environmental features on gene flow and genetic structure in *D. caspius*. Identifying locally adapted populations and fine-scale population structure is crucial in making knowledge-based decisions in conservation management.

Using the genotype-environment association methods implemented in Samßada we were able to determine candidate loci suggestive of localized adaptation [48]. To identify environmental-induced selection rather than genetic drift, Samßada performs logistic regression between the geographic distribution of genotypes in a SNP against the geographic distribution of an environmental variable, and it does so by testing one SNP at a time (not simultaneously). Should drift become a problematic confounding factor, we would observe that many genotypes across the genome (maybe all SNPs) show a strong correlation with a specific environmental variable. However, that was not the case as we identified 751 genotypes (corresponding to 4.3% of the total variation) significantly (*p* < 0.01) associated with one of the seven environmental variables. Furthermore, the clustering of the environmental variables did not overlap with the genetic clustering (Figure S2).

Out of the selected genotypes, 65 were located within coding regions (Figure S3). This seemingly lower variation in coding versus non-coding regions might be explained by a general higher number of SNPs present in non-coding parts of the genome. Furthermore, genome-wide functional studies have uncovered large amounts of functional elements in non-coding regions of the genome, which act as regulatory variations and include transcription factor binding sites or epigenetic modifications [68]. Our results showed that isothermality, annual temperature range, and annual mean temperature were the bioclimatic variables associated with most genotypes under selection (478, 127 and 87), respectively. Isothermality and annual precipitation were most informative in the PCA of the environmental variables (Figure S2). Isothermality refers to the magnitude of change between day-to-night temperature oscillations and summer-to-winter temperature oscillation [45]. This reinforces the hypothesis that increased mean year-to-year temperature variation due to climate change is likely to be problematic for snake populations in general and possibly negatively affect *D. caspius* populations [69]. Specifically, the limiting factor of *D. caspius* species distribution was described as the coldest temperature of the coldest month [17], which predicts that global warming may lead to the future range expansion of the species northwards. Because terrestrial ectotherms rely on behavioral thermoregulation [70] and are especially vulnerable to diurnal temperature fluctuations [71,72], it is not surprising that isothermality might have the highest number of associated genotypes. Adaptation to isothermal changes will be highly relevant to the survival of the species, as this environmental variable together with diurnal temperature range, is impacted by global warming [73].

Only two genotypes under selection were associated with annual precipitation. The amount of rainfall and the optimal (preferred) body temperature in ectotherms might be correlated as habitats with high rainfall provide worse conditions for behavioral thermoregulation [71]. However, this result might be explained in the context of *D. caspius*’ preferred habitat of warm and dry rocky areas [11,18,21] and the fact that across its distribution range, a considerable proportion of the precipitation falls in their hibernation period.

We attempted to identify whether genotypes under selection clustered geographically as it may reflect possible kinship between individuals and spatial variation in environmental variables. However, most selected genotypes located in coding regions were evenly distributed across all *D. caspius* populations (Figure S3), suggesting the important role that those genotypes have in the environmental adaptation potential of the species in general. Most of the genes harboring selected genotypes associated with isothermality and temperature had general functions related to neuronal development and differentiation, signal transduction, and encoding proteins of the calcium ion channels (Figure S3). A few rare genes were selected only in one or two populations, e.g., the gene DAGLA, associated with the average precipitation of the driest quarter in the Northern Hungarian (N-HU) and Samos (SAM) snakes, is required for axonal growth during development and for retrograde synaptic signaling at mature synapses [74]. Similarly, the genotype located in KIAA0408 (uncharacterized protein-coding gene) is associated with the mean temperature of the wettest quarter, and the selected genotype was present only in two individuals from Villány Hills (S-HU), as well as in the Bulgarian, Turkey (BALK-ANAT) and Samos (SAM) populations. The functional relationship between the identified genes and environmental variables, however, is currently unknown and would be an important avenue for further studies. It would certainly be informative to include additional individuals from these regions in further analyses to confirm the observed patterns and, in combination with additional genetic markers (e.g., from whole-genome re-sequencing), attempt to develop a deeper understanding of the evolutionary history and adaptive potential of these populations.

### 4.3. Conclusions and Conservation Management Recommendations

With a comprehensive data set of 17K genome-wide SNPs and samples covering a significant part of its global distribution range, we investigated phylogeography, gene flow, and adaptive selection in *D. caspius*. We attempted to distinguish between selection signals and demographic processes by employing multivariate analysis as implemented in the Samßada software. Limitations with the number of samples from each location did not allow us to test more specific models of demographic processes, e.g., based on coalescent analyses with approximate Bayesian computation. We are currently working on a sampling strategy to fill sampling gaps (in space and number), e.g., including locations north of the Danube River in southern Romania and along the Mediterranean coast, which might help us to understand the current high differentiation of the northern Hungarian (N-HU) population and to systematically follow the (re-) colonization routes of *D. caspius* into Central Europe after the last glacial period. This will also have an impact on conservation management recommendations: The results obtained here are indicative of a strong fragmentation in the species distribution range that has rendered the *D. caspius* populations relatively isolated from each other. It is important for this to be accounted for when considering moving animals across populations to reduce the effect of inbreeding or to increase population numbers. Furthermore, we found that 4.3% of the genotypes are associated with certain environmental variables and that the genetic diversity connected with such variation is widely distributed across the species range, thus suggesting that all populations harbor relevant adaptive genetic variation.

## Figures and Tables

**Table 1:** Information on the *D. caspius* individuals analyzed in this study. Population name, locality ID corresponding to Figure 1, Sample ID, sample origin, locality, country, and WGS 84 geocoordinates are given.

Population Name	Locality ID on Figure 1	Sample ID	Sample Origin	Locality	Country	Lat	Long
N-HU	1	HU_BU_HU_Gy697	Tissue (muscle)	Budapest, Hűvösvölgy	Hungary	47.5399	18.9661
	1	HU_BU_PV_Gy698	Tissue (muscle)	Budapest, Vöröskővár	Hungary	47.5561	18.9768
	1	HU_BU_PV_Gy838	Buccal swab	Budapest, Vöröskővár	Hungary	47.5561	18.9768
	1	HU_BU_PV_Gy925	Buccal swab	Budapest, Vöröskővár	Hungary	47.5562	18.9763
	1	HU_BU_PV_Gy955	Shed skin	Budapest, Vöröskővár	Hungary	47.5558	18.9767
	1	HU_BU_PV_Gy957	Buccal swab	Budapest, Vöröskővár	Hungary	47.5561	18.9768
	1	HU_BU_PV_Z003	Shed skin	Budapest, Vöröskővár	Hungary	47.5557	18.9752
DAN	2	HU_BU_SH_Gy693	Tissue (liver)	Budapest, Sas Hill	Hungary	47.4821	19.0196
	3	HU_BU_FH_Z024	Shed skin	Budapest, Farkas Hill	Hungary	47.4724	18.9427
	4	HU_DF_DU2	Blood	Dunaújváros	Hungary	46.9106	18.9461
	4	HU_DF_DUJ22	Blood	Dunaújváros	Hungary	46.9106	18.9461
	5	HU_DT_DF1	Blood	Dunaföldvár	Hungary	46.8027	18.9406
	6	HU_PT_PV2	Blood	Paks	Hungary	46.6626	18.8605
	6	HU_TO_PA_Z027	Shed skin	Paks	Hungary	46.6626	18.8605
	8	HR_BR_B01	Shed skin	Batina	Croatia	45.8334	18.8382
	8	HR_BR_B02	Shed skin	Batina	Croatia	45.8334	18.8387
S-HU	7	HU_VB_Sz1	Blood	Villány	Hungary	45.8571	18.4185
	7	HU_VB_Sz12	Blood	Villány	Hungary	45.8571	18.4185
	7	HU_VB_Sz13	Blood	Villány	Hungary	45.8571	18.4185
	7	HU_VB_Sz16	Blood	Villány	Hungary	45.8571	18.4185
	7	HU_VB_Sz17	Blood	Villány	Hungary	45.8571	18.4185
	7	HU_VB_Sz2	Blood	Villány	Hungary	45.8571	18.4185
	7	HU_VB_Sz6	Blood	Villány	Hungary	45.8571	18.4185
	7	HU_VB_Sz7	Blood	Villány	Hungary	45.8571	18.4185
	7	HU_VB_Sz8	Blood	Villány	Hungary	45.8571	18.4185
I-CR	9	HR_LA_Oi_O02	Blood	Olib island	Croatia	44.3656	14.7855
	10	HR_LA_PP_L11	Blood	Lastovo island	Croatia	42.7532	16.9169
BALK-ANAT	11	RS_ZL_Y5	Blood	Zlot	Serbia	44.0387	21.9300
	11	RS_ZL_Y6	Blood	Zlot	Serbia	44.0387	21.9300
	12	RS_BU_Y3	Blood	Brestovacka	Serbia	44.0621	22.0497
	19	BG_SO_1415	Shed skin	Sozopol	Bulgaria	42.3955	27.6996
	19	BG_SO_764	Tissue (muscle)	near Sozopol	Bulgaria	42.4104	27.6497
	20	GR_LO_763	Tissue (muscle)	Loutros	Greece	40.8806	26.0458
	22	TU_IZ_J57	Tissue (muscle)	Izmir	Turkey	38.4237	27.1428
C-BAL	13	RS_CU_1708	Blood	Cukarka	Serbia	42.2874	21.7082
	14	MK_Pi_1514	Tissue (muscle)	Pirava	North Macedonia	41.3080	22.5356
	15	MK_BK_1577	Tissue (muscle)	Bilbil Kamen	North Macedonia	41.0398	21.2997
	16	MK_PPj_1632	Tissue (muscle)	Pokrvenik, Prespansko jezero	North Macedonia	41.0150	20.9648
	17	AL_BO_721	Tissue (muscle)	Boboshticë	Albania	40.5505	20.7597
	18	AL_PE_1856	Tissue (muscle)	Pepellash	Albania	40.4619	20.6672
SAM	21	GR_SA_D19	Tissue (muscle)	Samos	Greece	37.7547	26.9777
	21	GR_SA_D8	Tissue (muscle)	Samos	Greece	37.7547	26.9777
CRI-BES	23	UA_BDT_1184	Tissue (scale)	Tabaky	Ukraine	45.7332	28.6020
	24	UA_PE_2384	Tissue (muscle)	Peredovoe	Ukraine /Crimea	44.5339	33.8254
	25	UA_MM_2382	Tissue (muscle)	Karadag Mt.	Ukraine /Crimea	44.9319	35.2212
	25	UA_KU_1185	Tissue (muscle)	Kurortnoe	Ukraine /Crimea	44.9181	35.2028
	25	UA_KU_1186	Tissue (muscle)	Kurortnoe	Ukraine /Crimea	44.9126	35.2006
	25	UA_KU_2383	Tissue (muscle)	Kurortnoe	Ukraine /Crimea	44.9103	35.1625
	25	UA_SK_1183	Tissue (muscle)	Schebetovka	Ukraine /Crimea	44.9496	35.1873
	26	UA_VU_2391	Tissue (scale)	Vulkanovka	Ukraine /Crimea	45.1503	35.9309
	27	UA_PT_2385	Tissue (scale)	Ptashkino	Ukraine /Crimea	45.1716	36.1635
	28	UA_YA_2386	Tissue (scale)	Yakovenkovo	Ukraine /Crimea	45.0451	36.2412
	29	UA_BO_2389	Tissue (scale)	Bondarenkovo	Ukraine /Crimea	45.4467	36.4346

Lat = latitude; Long = longitude in World Geodetic System (WGS) 84. Population names correspond to populations identified by PCA and visualized in Figure 1 and Figure 2.

**Table 2:** *D. caspius* population pairwise *F*_ST_ values.

Population	DAN	S-HU	N-HU	C-BAL	BALK-ANAT	CRI-BES
DAN	-					
S-HU	0.340 *	-				
N-HU	0.420 *	0.595 *	-			
C-BAL	0.401 *	0.565 *	0.261 *	-		
BALK-ANAT	0.132 *	0.344 *	0.321 *	0.302 *	-	
CRI-BES	0.295 *	0.487 *	0.465 *	0.458 *	0.194 *	-

*D. caspius* population pairwise *F*_ST_ values calculated with ARLECORE (v 3.5.2). Only those populations with more than five individuals were included. Significant values (*p* < 0.05, 10000 permutations) are indicated with an asterisk(*). Population names correspond to populations identified by PCA, described in Table 1, and visualized on Figure 1 and Figure 2.

## Data Availability

The quality controlled genotype data (.map and .ped) included in this study as well as the raw trimmed reads and RAD loci that STACKS used to call SNPs, alongside bioinformatics codes are available at DRYAD (doi:10.5061/dryad.mgqnk98xm).

## Supplementary Materials

The following are available online at https://www.mdpi.com/2073-4425/11/10/1218/s1, Figure S1: Workflow of the landscape genomic analysis with Samßada, Figure S2: Principal component analysis biplot of individuals and explanatory variables, Figure S3: Genes associated with environmental variables, Table S1: Sequence coverage for each individual at all 17518 SNPs, estimated using VCFtools 0.1.16, Table S2: Selected WorldClim environmental and bioclimatic variables and the values of the variables at the sample locations, Table S3: Nucleotide diversity and inbreeding coefficients, Table S4: Definitions of seven WordClim bioclimatic variables used in the study.
