# Supplementary material for: Landscape Genomics of a Widely Distributed Snake, Dolichophis caspius (Gmelin, 1789) across Eastern Europe and Western Asia
Source: Genes (Basel). 2020 Oct 17;11(10):1218. doi: 10.3390/genes11101218 (PMC7603136; doi:10.3390/genes11101218)
Supplement: Supplementary file 1 [file genes-11-01218-s001.zip › genes-934100/Mahtani-Williams_etal_Figure_S3_revised.pdf]

Supplementary Figure 3. Genes associated with environmental variables. Population names and individual codes are corresponding with Table 1. and Figure 1. Colors are only used for better visualization.

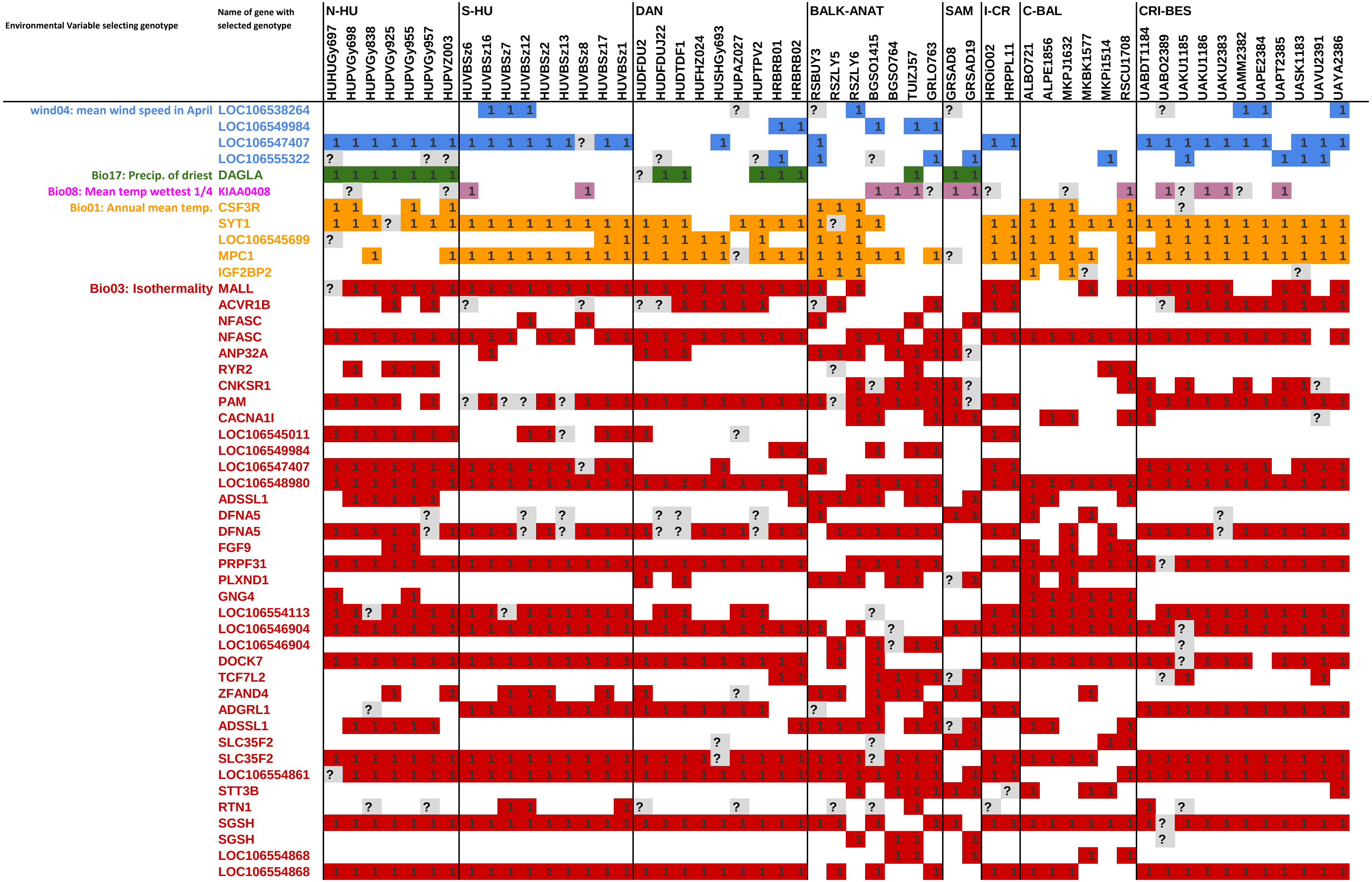

	Gene	HUHUGy697	HUPVGy698	HUPVGy838	HUPVGy925	HUPVGy955	HUPVGy957	HUPVZ003	HUVBSz6	HUVBSz16	HUVBSz7	HUVBSz12	HUVBSz2	HUVBSz13	HUVBSz8	HUVBSz17	HUVBSz1
	KCNH5	1	1	1	1	1	1	1	1	1	1	1	1	1	1	1	1
	RHEB	1	1	1	1	1	1	1	1	1	1	1	1	1	1	1	1
	BAIAP2L2										?						
	CACNA1S													1	1		1
Bio07: Annual Temp. Range	SDE2		1	1		1					?		?				
	APELA								1					1		1	
	CD34	1	1	1	1	1	1	1	1				1	1	1		1
	LOC106546708								1	1	1	1	1	1	1	1	1
	PCCA								1	1	1	1	1	1	1	1	1
	PAM	1	1	1	1		1		?	1	?	?	1	?	1	1	1
	CTNND1								1	1	1		1	1	1	1	1
	LOC106546904	1	1	1	1	1	1	1	1	1	1	1	1	1	1	1	1
	LOC106546904																
	HDAC11	1	1	1	1	1	1	1	1	1	1	1	1	1	1	1	1
	MYOM1							1		1	1				1	1	1
	LOC106543088	1	1	1	1	1	1	1	1	1	1	1	1	1	1	1	1
	RGS18	1	1	1	1	1	1	1									
		N-HU							S-HU								

Gene	HUDFDU2	HUDFDUJ22	HUDTDF1	HUFHZ024	HUSHGy693	HUPAZ027	HUPTPV2	HRBRB01	HRBRB02	RSBUY3	RSZLY5	RSZLY6	BGSO1415	BGSO764	TUIZJ57	GRLO763
KCNH5				1	1											
RHEB	1	1	1	1	1	1	1	1	1	1		1			1	
BAIAP2L2		1		1						1	1	1			1	
CACNA1S											1					
SDE2	1	1	1	1	1		1	1	1	1						
APELA																
CD34	1	1	?	1	?		1	1	1		?	1	1	1	1	1
LOC106546708		1											?			
PCCA	1	1	1	1	1	1	1	1	1	1		1		1	1	
PAM	1	1	1	1	1	1	1	1	1	1	?	1	1	1	1	1
CTNND1						1	1			1						
LOC106546904	1	1	1	1	1	1	1	1	1	1		1		?		
LOC106546904											1		1	?	1	1
HDAC11	1	1	1	1	1	1	1	1	1	1				1	1	
MYOM1																
LOC106543088	1	1	1	1	1	1	1	1	1	1	1	1		1	1	
RGS18	?				?					?		1				
	DAN									BALK-ANAT						

Gene	GRSAD8	GRSAD19	HROiO02	HRPPL11	ALBO721	ALPE1856	MKPJ1632	MKBK1577	MKPi1514	RSCU1708
KCNH5			1	1	1		1	1	1	1
RHEB			1	1	1		1	1		1
BAIAP2L2	1	1			1	1				1
CACNA1S	?	1			1	1	1			
SDE2			1	1	1	1		1	1	1
APELA					1			1		1
CD34	1	1	1	1	?					
LOC106546708			1	1	1	1	1	1	1	
PCCA									1	1
PAM	1	?	1	1						
CTNND1			1	1	1	1	1	1	1	
LOC106546904	1	1	1	1	1	1	1	1	1	1
LOC106546904										
HDAC11	1		1	1	1	1	1	1	1	1
MYOM1	1	1	1	1						
LOC106543088			1	1	1	1	1	1	1	1
RGS18	?	1	1	1			1			
	SAM		I-CR		C-BAL					

Gene	UABDT1184	UABO2389	UAKU1185	UAKU1186	UAKU2383	UAMM2382	UAPE2384	UAPT2385	UASK1183	UAVU2391	UAYA2386
KCNH5	1	?	1	1	1	1	1	1	1	1	1
RHEB	1	1	1	1	1	1	1	1	1	1	1
BAIAP2L2	1			1		1		1	1	?	
CACNA1S											
SDE2	1	1	1	1	1	1		1	1	?	?
APELA											
CD34	1	1	1	1	1	1	1	1	1	1	1
LOC106546708	1	?			1			1		1	
PCCA	1	1	1	1	1	1	1	1	1	1	1
PAM	1	1	1	1	1	1	1	1	1	1	1
CTNND1	1	1	1	1	1	1	1	1	1	1	1
LOC106546904	1	1	?	1	1	1	1	1	1	1	1
LOC106546904			?								
HDAC11	1	1	1	1	1	1	1	1	1	1	1
MYOM1											
LOC106543088	1	?	1	1	1	1	1	1	1	1	1
RGS18											
	CRI-BES										
