# Supplementary material for: Landscape Genomics of a Widely Distributed Snake, Dolichophis caspius (Gmelin, 1789) across Eastern Europe and Western Asia
Source: Genes (Basel). 2020 Oct 17;11(10):1218. doi: 10.3390/genes11101218 (PMC7603136; doi:10.3390/genes11101218)
Supplement: Supplementary file 1 [file genes-11-01218-s001.zip › genes-934100/Mahtani-Williams_etal_Supporting_Information_revised_corrected.docx]

**Supporting Information for:**

**Landscape genomics of a widely distributed snake (*Dolichophis caspius*, Gmelin, 1789) across eastern Europe and western Asia**

S. Mahtani-Williams, W. Fulton, A. Desvars-Larrive, S. Lado, J. P. Elbers, B. Halpern, D. Herczeg, G. Babocsay, B. Lauš, Z. T. Nagy, D. Jablonski, O. Kukushkin, P. Orozco-terWengel, J. Vörös, P. A. Burger

Corresponding authors:

J. Vörös: voros.judit@nhmus.hu

P. A. Burger: pamela.burger@vetmeduni.ac.at

**Supplementary Table 1.** Sequence coverage for each individual at all 17518 SNPs, estimated using VCFtools 0.1.16.

| Individual | Mininum | 1st_Quartile | Median | Mean | 3rd_Quartile | Maximum |
| --- | --- | --- | --- | --- | --- | --- |
| AL_BO_721 | 0 | 28 | 41 | 39.86 | 53 | 130 |
| AL_PE_1856 | 0 | 51 | 68 | 65.83 | 82 | 175 |
| BG_SO_1415 | 0 | 5 | 10 | 10.46 | 15 | 45 |
| BG_SO_764 | 0 | 21 | 29 | 28.71 | 37 | 97 |
| TU_KL_J57 | 0 | 50 | 68 | 64.25 | 81 | 196 |
| GR_LO_763 | 0 | 60 | 88 | 83.57 | 110 | 265 |
| GR_SA_D19 | 0 | 15 | 22 | 21.56 | 28 | 76 |
| GR_SA_D8 | 0 | 5 | 8 | 8.67 | 12 | 31 |
| HR_BR_B01 | 0 | 17 | 24 | 23.97 | 31 | 78 |
| HR_BR_B02 | 0 | 20 | 28 | 27.66 | 35 | 98 |
| HR_Oi_O02 | 0 | 15 | 27 | 30.27 | 43 | 124 |
| HR_PP_L11 | 0 | 9 | 15 | 16.29 | 23 | 69 |
| HU_DF_DU2 | 0 | 13 | 19 | 20.02 | 27 | 73 |
| HU_DF_DUJ22 | 0 | 42 | 58 | 57.82 | 74 | 172 |
| HU_DT_DF1 | 0 | 60 | 83 | 81.56 | 105 | 243 |
| HU_FH_Z024 | 0 | 35 | 46 | 45.09 | 57 | 149 |
| HU_HU_Gy697 | 0 | 7 | 12 | 13.60 | 19 | 70 |
| HU_PA_Z027 | 0 | 8 | 12 | 12.71 | 17 | 48 |
| HU_PT_PV2 | 0 | 36 | 52 | 52.41 | 68 | 194 |
| HU_PV_Gy698 | 0 | 37 | 50 | 48.82 | 62 | 145 |
| HU_PV_Gy838 | 0 | 7 | 13 | 13.45 | 19 | 62 |
| HU_PV_Gy925 | 0 | 50 | 74 | 70.66 | 93 | 242 |
| HU_PV_Gy955 | 0 | 12 | 17 | 18.33 | 24 | 74 |
| HU_PV_Gy957 | 0 | 10 | 16 | 16.91 | 23 | 61 |
| HU_PV_Z003 | 0 | 16 | 22 | 21.83 | 28 | 69 |
| HU_SH_Gy693 | 0 | 69 | 91 | 88.20 | 111 | 240 |
| HU_VB_Sz1 | 0 | 26 | 37 | 38.38 | 50 | 135 |
| HU_VB_Sz12 | 0 | 36 | 49 | 49.36 | 63 | 156 |
| HU_VB_Sz13 | 0 | 21 | 31 | 31.80 | 42 | 118 |
| HU_VB_Sz16 | 0 | 23 | 33 | 33.88 | 44 | 126 |
| HU_VB_Sz17 | 0 | 54 | 73 | 70.39 | 89 | 242 |
| HU_VB_Sz2 | 0 | 24 | 35 | 36.51 | 48 | 135 |
| HU_VB_Sz6 | 0 | 27 | 39 | 39.50 | 51 | 133 |
| HU_VB_Sz7 | 0 | 26 | 37 | 37.34 | 49 | 122 |
| HU_VB_Sz8 | 0 | 24 | 36 | 36.95 | 49 | 129 |
| MK_BK_1577 | 0 | 38 | 53 | 51.82 | 67 | 133 |
| MK_PJ_1632 | 0 | 35 | 47 | 45.94 | 58 | 125 |
| MK_Pi_1514 | 0 | 15 | 21 | 20.95 | 27 | 69 |
| RS_BU_Y3 | 0 | 6 | 10 | 9.85 | 14 | 44 |
| RS_CU_1708 | 0 | 52 | 76 | 73.36 | 97 | 208 |
| RS_ZL_Y5 | 0 | 38 | 55 | 56.48 | 75 | 226 |
| RS_ZL_Y6 | 0 | 63 | 84 | 80.62 | 101 | 240 |
| UA_BDT_1184 | 0 | 71 | 104 | 97.96 | 129 | 312 |
| UA_BO_2389 | 0 | 6 | 9 | 10.00 | 14 | 44 |
| UA_KU_1185 | 0 | 13 | 21 | 21.56 | 30 | 87 |
| UA_KU_1186 | 0 | 41 | 59 | 57.02 | 74 | 174 |
| UA_KU_2383 | 0 | 46 | 68 | 67.59 | 90 | 195 |
| UA_MM_2382 | 0 | 80 | 115 | 109.53 | 145 | 362 |
| UA_PE_2384 | 0 | 39 | 53 | 51.94 | 65 | 153 |
| UA_PT_2385 | 0 | 38 | 58 | 56.26 | 76 | 182 |
| UA_SK_1183 | 0 | 40 | 53 | 51.31 | 64 | 145 |
| UA_VU_2391 | 0 | 5 | 8 | 8.41 | 12 | 31 |
| UA_YA_2386 | 0 | 54 | 78 | 74.23 | 98 | 268 |
| ALL_IND | 0 | 18 | 36 | 43.43 | 63 | 362 |

**Supplementary Table 2..** Selected WorldClim environmental and bioclimatic variables and the values of the variables at the sample locations, extracted from the raster dataset (*extract* function in the R package *raster*). Wind04 = wind speed April (m s-1), bio01 = Annual mean temperature (°C), Bio03 = Isothermality (percent), Bio07 = Temperature annual range(°C),  Bio08 = Mean temperature of wettest quarter (°C), Bio12 = Annual precipitation (mm), Bio17 = Precipitation of the driest quarter (mm).

| **ddRAD_code** | **longitude** | **latitude** | **wind04** | **bio01** | **bio03** | **bio07** | **bio08** | **bio12** | **bio17** |
| --- | --- | --- | --- | --- | --- | --- | --- | --- | --- |
| HR_Oi_O02 | 14.785513 | 44.365657 | 3.099999905 | 15.29583335 | 26.60116576 | 22.89999962 | 16.0666666 | 899 | 147 |
| HR_PP_L11 | 16.916964 | 42.753214 | 3.799999952 | 16.63750001 | 27.55319098 | 23.50000095 | 10.73333311 | 678 | 84 |
| RS_BU_Y3 | 22.04976 | 44.06213 | 2.799999952 | 10.03333335 | 32.32484042 | 31.40000057 | 14.40000025 | 664 | 130 |
| RS_ZL_Y5 | 21.93003 | 44.03877 | 3.5 | 7.516666732 | 31.37254914 | 27.20000029 | 14.48333327 | 729 | 137 |
| RS_ZL_Y6 | 21.93003 | 44.03877 | 3.5 | 7.516666732 | 31.37254914 | 27.20000029 | 14.48333327 | 729 | 137 |
| HU_DF_DU2 | 18.94615 | 46.9106 | 3.200000048 | 10.77083337 | 28.3862431 | 31.50000095 | 19.10000038 | 527 | 84 |
| HU_DF_DUJ22 | 18.94615 | 46.9106 | 3.200000048 | 10.77083337 | 28.3862431 | 31.50000095 | 19.10000038 | 527 | 84 |
| HU_DT_DF1 | 18.94062 | 46.8027 | 3.200000048 | 10.81666672 | 28.32800758 | 31.30000067 | 19.13333352 | 524 | 83 |
| HU_FH_Z024 | 18.94272 | 47.47241 | 3.299999952 | 10.3250001 | 27.64900618 | 30.20000076 | 18.43333371 | 564 | 98 |
| HU_PA_Z027 | 18.86053 | 46.6626 | 3.200000048 | 10.82500003 | 28.5410005 | 31.30000067 | 19.10000038 | 533 | 82 |
| HU_PT_PV2 | 18.86053 | 46.6626 | 3.200000048 | 10.82500003 | 28.5410005 | 31.30000067 | 19.10000038 | 533 | 82 |
| HU_SH_Gy693 | 19.019626 | 47.482149 | 3.099999905 | 11.05000008 | 27.09790177 | 28.60000086 | 18.90000025 | 537 | 92 |
| HR_BR_B01 | 18.838241 | 45.83343 | 3 | 10.87916671 | 30.26024383 | 30.09999943 | 20.26666673 | 598 | 100 |
| HR_BR_B02 | 18.838762 | 45.833452 | 3 | 10.87916671 | 30.26024383 | 30.09999943 | 20.26666673 | 598 | 100 |
| HU_VB_Sz6 | 18.418572 | 45.857184 | 3.099999905 | 10.71250018 | 29.93929334 | 30.20000076 | 18.56666708 | 637 | 106 |
| HU_VB_Sz16 | 18.418572 | 45.857184 | 3.099999905 | 10.71250018 | 29.93929334 | 30.20000076 | 18.56666708 | 637 | 106 |
| HU_VB_Sz7 | 18.418572 | 45.857184 | 3.099999905 | 10.71250018 | 29.93929334 | 30.20000076 | 18.56666708 | 637 | 106 |
| HU_VB_Sz12 | 18.418572 | 45.857184 | 3.099999905 | 10.71250018 | 29.93929334 | 30.20000076 | 18.56666708 | 637 | 106 |
| HU_VB_Sz2 | 18.418572 | 45.857184 | 3.099999905 | 10.71250018 | 29.93929334 | 30.20000076 | 18.56666708 | 637 | 106 |
| HU_VB_Sz13 | 18.418572 | 45.857184 | 3.099999905 | 10.71250018 | 29.93929334 | 30.20000076 | 18.56666708 | 637 | 106 |
| HU_VB_Sz8 | 18.418572 | 45.857184 | 3.099999905 | 10.71250018 | 29.93929334 | 30.20000076 | 18.56666708 | 637 | 106 |
| HU_VB_Sz17 | 18.418572 | 45.857184 | 3.099999905 | 10.71250018 | 29.93929334 | 30.20000076 | 18.56666708 | 637 | 106 |
| HU_VB_Sz1 | 18.418572 | 45.857184 | 3.099999905 | 10.71250018 | 29.93929334 | 30.20000076 | 18.56666708 | 637 | 106 |
| HU_HU_Gy697 | 18.966192 | 47.539936 | 3.299999952 | 10.05833324 | 27.51937972 | 30.09999943 | 18.34999975 | 563 | 98 |
| HU_PV_Gy698 | 18.976898 | 47.55616 | 3.299999952 | 9.970833314 | 27.56622532 | 30.19999933 | 18.2166667 | 568 | 100 |
| HU_PV_Gy838 | 18.976898 | 47.55616 | 3.299999952 | 9.970833314 | 27.56622532 | 30.19999933 | 18.2166667 | 568 | 100 |
| HU_PV_Gy925 | 18.97637 | 47.55624 | 3.299999952 | 9.970833314 | 27.56622532 | 30.19999933 | 18.2166667 | 568 | 100 |
| HU_PV_Gy955 | 18.9767 | 47.55585 | 3.299999952 | 9.970833314 | 27.56622532 | 30.19999933 | 18.2166667 | 568 | 100 |
| HU_PV_Gy957 | 18.976898 | 47.55616 | 3.299999952 | 9.970833314 | 27.56622532 | 30.19999933 | 18.2166667 | 568 | 100 |
| HU_PV_Z003 | 18.97527 | 47.55578 | 3.299999952 | 9.970833314 | 27.56622532 | 30.19999933 | 18.2166667 | 568 | 100 |
| AL_BO_721 | 20.759779 | 40.550552 | 2.200000048 | 10.41249997 | 34.89914968 | 31.40000057 | 7.100000011 | 817 | 120 |
| AL_PE_1856 | 20.66722 | 40.46194 | 2.099999905 | 10.53750003 | 34.79381506 | 29.0999999 | 7.51666681 | 888 | 120 |
| MK_PPj_1632 | 20.96488 | 41.015 | 2.5 | 10.49583338 | 33.70949175 | 28.79999924 | 7.049999936 | 766 | 123 |
| MK_BK_1577 | 21.29977 | 41.03988 | 2.5 | 11.0125001 | 33.51063863 | 32.89999914 | 6.583333453 | 649 | 115 |
| MK_Pi_1514 | 22.5356 | 41.30806 | 2 | 14.31250009 | 33.30889616 | 34.0999999 | 9.366666873 | 435 | 88 |
| RS_CU_1708 | 21.70822 | 42.28746 | 2.599999905 | 11.06666662 | 34.03193681 | 33.39999914 | 15.36666656 | 551 | 111 |
| BG_SO_1415 | 27.69961 | 42.39552 | 3.299999952 | 12.83749994 | 30.46085752 | 26.40000039 | 9.849999825 | 543 | 102 |
| BG_SO_764 | 27.6497 | 42.41049 | 3.299999952 | 12.86249997 | 31.14197459 | 27.00000039 | 9.683333238 | 539 | 102 |
| TU_IZ_J57 | 27.14282 | 38.42373 | 3 | 17.54583333 | 32.88849363 | 28.10000086 | 10.79999995 | 675 | 16 |
| GR_LO_763 | 26.04588 | 40.88061 | 2.799999952 | 14.14999996 | 30.85748799 | 27.6 | 7.350000047 | 559 | 60 |
| GR_SA_D8 | 26.97777 | 37.75478 | 3.5 | 17.43333354 | 35.03546157 | 23.5 | 10.95000005 | 688 | 9 |
| GR_SA_D19 | 26.97777 | 37.75478 | 3.5 | 17.43333354 | 35.03546157 | 23.5 | 10.95000005 | 688 | 9 |
| UA_BDT_1184 | 28.6020806 | 45.733225 | 3.700000048 | 10.55833338 | 28.51405633 | 33.19999981 | 19.61666663 | 497 | 98 |
| UA_BO_2389 | 36.43468 | 45.44679 | 4.099999905 | 11.20833334 | 24.34210505 | 30.40000057 | 21.96666686 | 412 | 88 |
| UA_KU_1185 | 35.2028361 | 44.9181417 | 3.799999952 | 11.8666667 | 25.88168401 | 29.30000043 | 4.466666579 | 489 | 111 |
| UA_KU_1186 | 35.2006694 | 44.9126528 | 3.799999952 | 12.22083336 | 25.79635958 | 29.29999971 | 22.33333333 | 479 | 107 |
| UA_KU_2383 | 35.1625 | 44.91036 | 3.799999952 | 11.82500002 | 25.94501756 | 29.0999999 | 4.416666557 | 497 | 112 |
| UA_MM_2382 | 35.22121 | 44.9319 | 4 | 11.10833326 | 24.38162597 | 28.29999924 | 3.833333353 | 521 | 120 |
| UA_PE_2384 | 33.82542 | 44.5339 | 3.900000095 | 9.129166653 | 25.63868588 | 27.4000001 | 1.866666655 | 641 | 137 |
| UA_PT_2385 | 36.16354 | 45.17169 | 3.599999905 | 11.32083327 | 28.6507936 | 31.50000048 | 21.86666663 | 395 | 86 |
| UA_SK_1183 | 35.1873528 | 44.949625 | 3.799999952 | 11.68749997 | 27.27521977 | 30.39999914 | 3.949999984 | 494 | 111 |
| UA_VU_2391 | 35.93095 | 45.15032 | 3.599999905 | 11.18749995 | 29.4444444 | 31.50000048 | 21.66666651 | 418 | 93 |
| UA_YA_2386 | 36.24125 | 45.04512 | 3.900000095 | 11.55416666 | 25.39548008 | 29.50000048 | 21.88333337 | 380 | 85 |

**Supplementary Table 3.** Nucleotide diversity **(**average over loci**)** and inbreeding coefficients (FIS values of six Caspian Whipsnake populations (n > 5), calculated with ARLECORE (v 3.5.2) using 10000 permutations.

| **Population**  **(n individuals)** | **Nucleotide diversity**  +/- SD | **FIS** | ***P* (Rand FIS >= Obs FIS)** |
| --- | --- | --- | --- |
| N-HU (7) | 0.108 +/- 0.055 | -0.106 | 0.849 |
| S-HU (9) | 0.066 +/- 0.033 | 0.027 | 0.346 |
| DAN (9) | 0.131 +/- 0.065 | 0.446 | 0.000 |
| BALK-ANAT (7) | 0.167 +/- 0.085 | 0.095 | 0.078 |
| C-BAL (6) | 0.158 +/- 0.082 | 0.121 | 0.042 |
| CRI-BES (11) | 0.110 +/- 0.054 | 0.069 | 0.085 |

**Supplementary Table 4.** Definitions of seven WordClim bioclimatic variables used in the study [53].

| Variable | Definition |
| --- | --- |
| Bio 1—Annual Mean Temperature | The annual mean temperature |
| Bio 3—Isothermality | Isothermality quantifies how large the day-to-night temperatures oscillate relative to the summer-to-winter (annual) oscillations. |
| Bio 7—Annual Temperature Range | A measure of temperature variation over a given period. |
| Bio 8—Mean Temperature of Wettest Quarter | This quarterly index approximates mean temperatures that prevail during the wettest season. |
| Bio 12—Annual Precipitation | This is the sum of all total monthly precipitation values. |
| Bio 17—Precipitation of Driest Quarter | This quarterly index approximates total precipitation that prevails during the driest quarter. |

**Supplementary Figure 1**. Workflow of the landscape genomic analysis with Samßada.


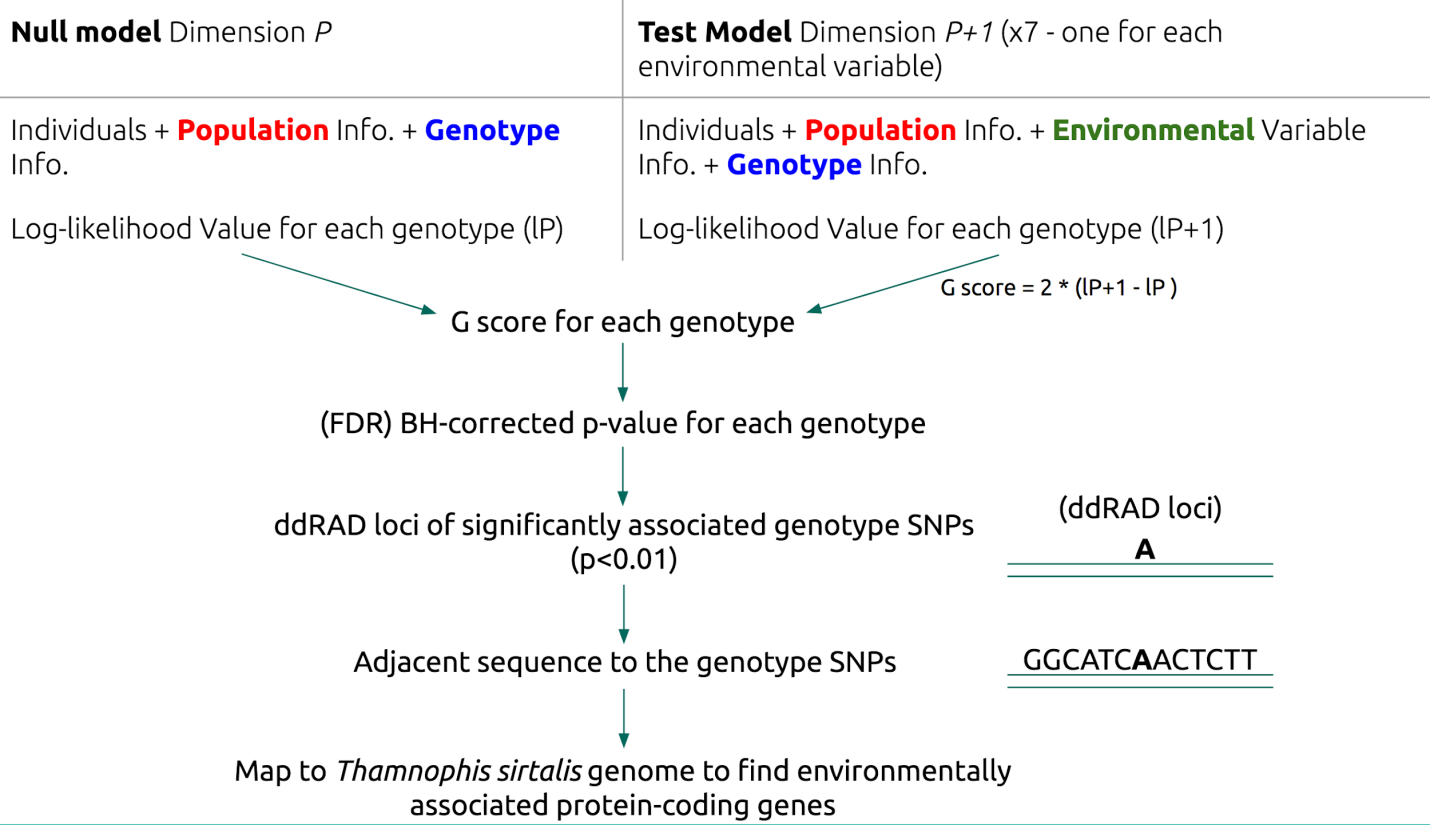


**Supplementary Figure 2.** Principal component analysis (PCA) biplot of individuals (n = 53) and explanatory variables (n = 7). The biplot shows the PCA scores of the explanatory variables as vectors (in black) and individuals (colored dots) of each population: southern Hungary (S-HU), the Danube region (DAN) of Hungary and Croatia, Balkan-Anatolia including western Turkey, Bulgaria, Greece and Serbia (BALK-ANAT), the Greek island Samos (SAM) and the Northern Black Sea region including the Crimean Peninsula and Bessarabia in Ukraine (CRI-BES) from those in northern Hungary (N-HU), Dalmatian Archipelago of Croatia (I-CR), and the central Balkans in the limits of Albania and Republic of North Macedonia (C-BAL), of the first (x-axis) and second (y-axis) principal components (PCs). Individuals on the same side as a given variable should be interpreted as having a high contribution on it. The magnitude of the vectors (lines) shows the strength of their contribution to each PC. Vectors pointing in similar directions indicate positively correlated variables, vectors pointing in opposite directions indicate negatively correlated variables, and vectors at proximately right angles indicate low or no correlation. Colored ellipses (size determined by a 0.95-probability level) show the observations grouped by population. In each population, the mean point (barycenter) is displayed as a bigger dot.


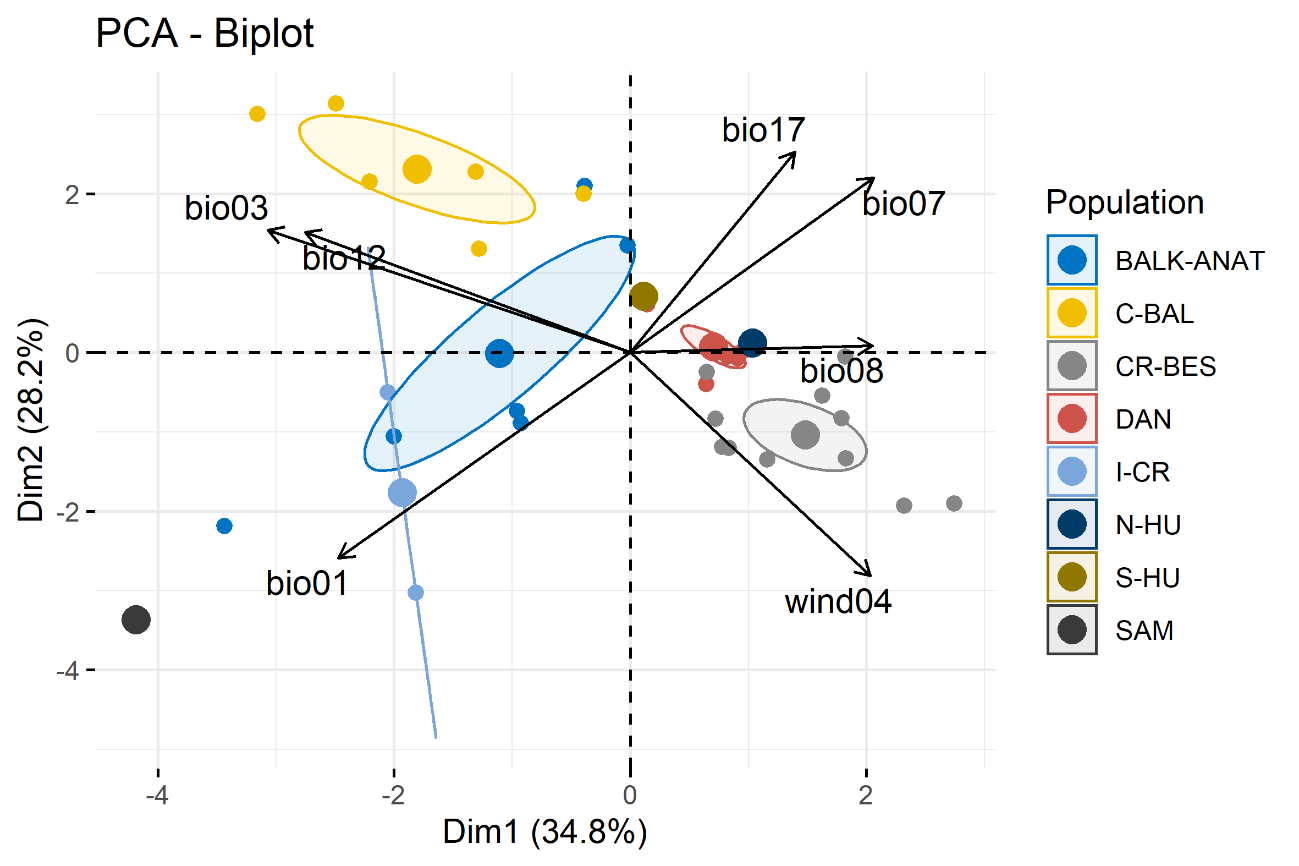


me
